# Evaluation of miglustat as maintenance therapy after enzyme therapy in adults with stable type 1 Gaucher disease: a prospective, open-label non-inferiority study

**DOI:** 10.1186/1750-1172-7-102

**Published:** 2012-12-27

**Authors:** Timothy M Cox, Dominick Amato, Carla EM Hollak, Cecile Luzy, Mariabeth Silkey, Ruben Giorgino, Robert D Steiner

**Affiliations:** 1 University of Cambridge, Addenbrooke's Hospital, Cambridge, UK; 2 Mount Sinai Hospital, University of Toronto, Toronto, Canada; 3 Academic Medical Centre, Amsterdam, The Netherlands; 4 Actelion Pharmaceuticals Ltd, Allschwil, Switzerland; 5 Child Development and Rehabilitation Center/Doernbecher Children’s Hospital, Oregon Health & Science University, Portland, OR, USA

**Keywords:** Gaucher disease, Miglustat, Enzyme therapy, Maintenance, Efficacy, Safety

## Abstract

**Background:**

Previous studies have provided equivocal data on the use of miglustat as maintenance therapy in Gaucher disease type 1. We report findings from a clinical trial evaluating the effects of miglustat treatment in patients with stable type 1 Gaucher disease after enzyme therapy.

**Methods:**

Adult type 1 Gaucher disease patients stabilized during at least 3 years of previous enzyme therapy were included in this 2-year, prospective, open-label non-inferiority study. The primary endpoint was percent change from baseline in liver volume. Secondary endpoints included changes in spleen volume, hemoglobin concentration and platelet count.

**Results:**

Forty-two patients were enrolled (mean±SD age, 45.1±12.7 years; previous enzyme therapy duration 9.5±4.0 years). Median (range) exposure to miglustat 100 mg t.i.d. was 658 (3–765) days. Twenty-one patients discontinued treatment prematurely; 13 due to adverse events, principally gastrointestinal. The upper 95% confidence limit of mean percent change in liver volume from baseline to end of treatment was below the non-inferiority margin of 10% (–1.1%; 95%CI −6.0, 3.9%). Mean (95%CI) changes in spleen volume, hemoglobin concentration and platelet count were 102 (24,180) mL, –0.95 (−1.38, –0.53) g/dL and −44.1 (–57.6, –30.7) ×10^9^/L, respectively.

**Conclusions:**

The primary efficacy endpoint was met; overall there was no change in liver volume during 24 months of miglustat therapy. Several patients showed a gradual deterioration in some disease manifestations, suggesting that miglustat could maintain clinical stability, but not in all patients. Miglustat demonstrated a predictable profile of safety and tolerability that was consistent with that reported in previous clinical trials and experience in clinical practice.

**Trial registration:**

Clinicaltrials.gov identifier NCT00319046

## Introduction

Type 1 Gaucher disease is an inherited lysosomal disorder caused by impaired activity of β-glucocerebrosidase, and xaccumulation of glucosylceramide in pathologic macrophages in the liver, spleen, bone marrow and, less frequently, the lungs [1,2]. The most common clinical variant is type 1 Gaucher disease, which is characterized by anemia, thrombocytopenia, hepatosplenomegaly and skeletal manifestations such as bone pain, osteopenia, fragility fractures and osteonecrosis [2-5].

Enzyme therapy (often mistakenly termed enzyme replacement therapy) with recombinant human β-glucocerebrosidase (imiglucerase [Cerezyme®], Genzyme Therapeutics; velaglucerase [VPRIV™], Shire plc) reduces organomegaly and improves hematologic and biochemical parameters in type 1 Gaucher disease [6,7]. However, enzyme therapy requires regular intravenous infusions, which are a lifestyle burden for some patients [8,9].

Miglustat (Zavesca®; Actelion Pharmaceuticals) is an orally active iminosugar which reversibly inhibits UDP-glucosylceramide synthase – the enzyme that catalyzes the rate-limiting step in glycosphingolipid biosynthesis. Miglustat was approved in Europe in 2002 and in the USA in 2003 for use as a substrate reduction therapy in adult patients with mild-to-moderate type 1 Gaucher disease for whom enzyme therapy is unsuitable or not a therapeutic option [10,11].

During developmental clinical trials, miglustat induced sustained reductions of liver and spleen volumes, with increased hemoglobin concentrations and platelet counts; the plasma activity of the biomarker, chitotriosidase, also decreased [12-15]. Similar effects have been observed in ‘real-world’ clinical practice settings [16,17]. It has also been suggested that miglustat improves cortical and trabecular bone mineral density (BMD) during 6 months to 2 years of therapy, and can reduce the frequency of bone pain in a sustained manner [18].

A randomized, open-label Phase II clinical trial evaluated the use of miglustat, given alone or in combination with enzyme therapy, in type 1 Gaucher disease patients stabilized by long-term treatment with imiglucerase [19]. However, equivocal beneficial effects on the disease, observed over a limited duration of assessment in a small number of patients, did not allow a definitive conclusion as to whether oral miglustat is effective in maintaining clinical stability in patients with type 1 Gaucher disease after they have received enzyme therapy. Further clinical trial data were requested by the European Medicines Association (EMA) to investigate this issue more fully, and a suitable study design was defined through subsequent consultation.

Here we report the findings of a study of miglustat as maintenance treatment in adult patients whose type 1 Gaucher disease had been controlled by enzyme therapy. As the inclusion of a placebo or non-drug arm was considered unethical for this patient population, the study was of an open-label, non-controlled design.

## Methods

### Study design and patients

This was a multicenter, international, open-label, Phase IIIb non-inferiority study comprising a 6-month screening period, a 24-month treatment period and a 28-day safety follow-up period. The primary objective was to evaluate the long-term effect of miglustat maintenance therapy on liver volume in adult patients with stable type 1 Gaucher disease who switched to miglustat from enzyme therapy. The secondary objectives were to examine the effects of miglustat on spleen volume and other type 1 Gaucher disease manifestations during long-term maintenance therapy. The safety and tolera-bility of miglustat was also assessed, including neurological and neuropsychological assessments.

Male and female patients aged at least 18 years with confirmed, stable type 1 Gaucher disease, who had undergone 3 or more years of enzyme therapy and had been on a stable enzyme dose for at least 6 months were included. Patients were required to have stable disease for 2 or more years before enrolment, defined by assessments during at least two appropriate time points confirming the following: stable organomegaly, with the last three liver and spleen volume assessments within 10% of the mean of these values (as assessed by magnetic resonance imaging [MRI] or computed X-ray tomography [CT]); a documented absence of symptomatic bone disease; mean hemoglobin level >11 g/dL; mean platelet count >100 × 10^9^/L; stable chitotriosidase activity, with the last three values within 20% of these values.

The stability of disease in individual study participants was adjudicated at the beginning of the study by an independent Steering Committee comprising five clinical experts in Gaucher disease who were neither investigators nor directly involved in the conduct of the study. This Steering Committee also adjudicated cases of suspected disease worsening during the study, and carried out a final adjudication of all referred cases at the end of the study.

Patients were excluded if they had manifestations of neuronopathic Gaucher disease, were non-ambulatory or had symptomatic bone disease. Other reasons for exclusion included splenectomy before 18 years of age, documented peripheral polyneuropathy comprising suggestive symptoms, compatible clinical signs and electrodiagnosis, a history of lactose intolerance, diarrhea (>3 liquid stools per day for >7 days), or a history of gastrointestinal disorders.

All patients provided written informed consent for study entry. The protocol and management structure were reviewed and approved by Institutional Review Boards or Independent Ethics Committees, and the study was conducted in accordance with the principles and procedures outlined in the Declaration of Helsinki. This trial was registered on http://www.clinicaltrials.gov on April 26, 2006 (identifier # NCT00319046).

### Treatment

All patients were prescribed oral miglustat 100 mg capsules three times daily throughout the 24-month treatment period, as specified in the prescribing information [11]. Dose reduction to 100 mg once or twice a day was permitted if patients experienced treatment-related diarrhea or other gastrointestinal symptoms, and this reduction was maintained until the symptoms subsided.

### Efficacy endpoints

The primary efficacy endpoint was the mean percent change in liver volume from baseline to Month 24. Liver volume was assessed by MRI every 6 months. Mean change in spleen volume from baseline to Month 24 was determined as a secondary efficacy endpoint. Spleen and liver volume were measured centrally by a specialized imaging center (SYNARC, Sèvres, France), as well as locally at each study center. Hemoglobin concentration and platelet count were also assessed locally during each clinic visit as secondary efficacy endpoints. The disease biomarker, chitotriosidase activity, was measured at a central laboratory in plasma samples obtained at all visits during the treatment period.

Mean change in the bone marrow burden (BMB) score from baseline to Month 24 was measured as a secondary efficacy endpoint based on the sum of scores from standardized semi-quantitative MRI-based assessments of bone marrow infiltration in axial (lumbar spine) and peripheral (femoral) sites at baseline, Month 12 and Month 24. Scores from 1 to 8 were assigned at each skeletal site, giving a maximum total of 16; higher scores represent more severe bone marrow infiltration. BMB scores were assessed locally at each study center (when possible), as well as centrally by a specialized imaging center (SYNARC) using published methods [20].

The proportion of patients that worsened was also determined. If investigators suspected disease worsening, the Steering Committee was asked to adjudicate as to whether, on the basis of the available clinical data, the patient had worsening of their disease. The following pre-defined criteria were taken as potential signals of disease worsening: increase in liver volume ≥10% from baseline; increase in spleen volume ≥10% from baseline; hemoglobin concentration decreased ≥1 g/dL from baseline; reduction in platelet count at two consecutive laboratory evaluations (according to pre-defined criteria); any clinical signs or symptoms (e.g. documented bone crisis) or change in relevant biomarkers (e.g. increase in chitotriosidase activity or changes in serum ferritin, ACE or TRAP) that suggested disease worsening in the investigator’s opinion. Confirmation of disease worsening by the Steering Committee required consistent clinical and biological documentation so that an isolated signal may not have been considered sufficient to confirm disease worsening; agreement in each case was reached by consensus.

When disease worsening was confirmed by the Steering Committee, miglustat was discontinued and enzyme therapy was reintroduced at a dose considered most appropriate by the investigator. Patients who discontinued treatment before Month 24 were allowed to remain in the study and underwent the remaining assessments. In cases of ‘suspected disease worsening’, where worsening of a patient’s disease was suspected by the Steering Committee but not confirmed by further clinical evaluation, additional information and/or assessments were requested. Under these circumstances, miglustat was continued until a final decision was reached. In cases where worsening of the disease was suspected by the investigator but not confirmed by the Steering Committee, treatment with miglustat could be continued until Month 24. A final adjudication on disease worsening based on all available data (including central laboratory imaging findings) was carried out at the end of the study, and the reported numbers of patients with clinical worsening is based on this final adjudication.

### Additional analysis: maintenance of therapeutic goals

An additional analysis of the achievement of accepted type 1 Gaucher disease therapeutic goals in patients who completed 24 months of miglustat treatment was also conducted. While not defined *a priori*, this analysis did not formally constitute a *post-hoc* analysis as it was included in the statistical analysis plan before database closure. Therapeutic goals were defined as previously described [21].

### Safety and tolerability

Treatment-emergent adverse events (i.e. all adverse events occurring in enrolled patients who received at least one dose of study medication) and serious adverse events were recorded throughout the study. Standard laboratory measurements including hemoglobin, platelet counts, biochemistry (including vitamins B_12_ and B_1_), and serum protein electrophoresis were examined in blood samples taken at each clinic visit.

Rigorous testing for peripheral neuropathy was performed in all patients by a trained, certified neurologist at each clinical site based on patients’ medical history and assessments of clinical signs and symptoms, as described previously [4]. All patients also underwent electrodiagnostic assessments at baseline, including nerve conduction velocity testing and electromyography [22-24]. If a patient presented new or aggravated clinical signs and/or symptoms that suggested possible peripheral neuropathy during the study, a repeat electrodiagnostic examination was carried out by the local neurologist to confirm the diagnosis. At the end of the study, electrodiagnostic data were evaluated centrally by an independent assessor. Diagnoses of peripheral neuropathy were assigned on the basis of compatible clinical signs and/or symptoms accompanied by compatible electrodiagnostic findings.

Neuropsychological assessments of cognitive function were also conducted by qualified personnel at baseline and every 6 months throughout the study. These comprised a battery of tasks derived from the CDR computerized cognitive assessment system, and assessed the following cognitive domains: simple reaction time; digit vigilance; choice reaction time; spatial working memory; numeric working memory; word recognition; picture recognition; Morse tapping [25,26]. Alertness and self-rated mood were also each evaluated using Bond-Lader visual analogue scales [27].

### Statistical analysis

A sample size of 42 patients was agreed as appropriate to meet the objectives of the study with the European Medicines Agency (EMA) Committee for Medicinal Products for Human Use (CHMP). Relevant study populations were defined as follows: ‘all-enrolled population’ - all enrolled patients regardless of whether they received study drug; ‘full analysis population’ - all enrolled patients who received study drug and had ≥1 post-baseline value for the primary endpoint; ‘per protocol population’ - patients in the full analysis population who completed the 24-month treatment period and did not have major protocol deviations that might have affected the primary endpoint.

Based on the standard deviation (SD) of the percent change in liver volume during a previous randomized clinical trial with miglustat (9.2%) [19], a clinically relevant non-inferiority limit of a 10% increase in liver volume was selected for the primary endpoint of this study. The null hypothesis was that the mean change in liver volume from baseline to Month 24 was ≥10%. The alternative hypothesis was that there was no change in liver volume. The null hypothesis was rejected if the upper limit of the two-sided 95% confidence interval (CI) around the mean change in liver volume from baseline to Month 24 was <10%.

Mean percent change in liver volume was determined by analyzing all available liver volume data from the full analysis population using a one-sample *t*-test with no adjustments for covariates. For the 20 patients in the full analysis population who discontinued miglustat prematurely, the worst (greatest) post-baseline value for liver volume on treatment was carried forward to substitute the Month 24 value in an ‘end of treatment’ analysis.

A sensitivity analysis was performed by comparing results from the full analysis population with the same analyses applied to the per-protocol population.

No formal statistical testing was carried out for secondary efficacy endpoints (change in spleen volume, hemoglobin concentration and platelet count), as there was no hypothesis test defined *a priori* for any of these endpoints, which are hypothesis-generating as opposed to inferential. Absolute values at baseline and follow up, and change from baseline data were summarized from the full analysis population using mean, SD and 95% CI. Descriptive ‘end of treatment’ analyses were conducted using last observations carried forward to impute missing values from patients who discontinued miglustat prematurely. Changes in hematological parameters and organ volumes were compared to previously defined therapeutic goals for Gaucher disease (21). These goals have not been validated but they may serve as benchmarks for clinically important changes.

## Results

### Patients

Forty-two patients (22 male, 20 female) were enrolled from 21 February 2006 onwards from 16 centers in Australia, Brazil, Canada, Czech Republic, France, Netherlands, Spain, Taiwan, UK, and the USA. The trial ended on 22 June 2010. The mean (SD) age at enrolment was 45.1 (12.7) years, and age at diagnosis was 24.8 (15.0) years (Table 1). Patients had previously received enzyme therapy for an average of 9.5 years, and had received the same dose of enzyme therapy (median [range] 54.7 [5.2–130.7] IU/kg/month) for approximately 5 years.

**Table 1:** Patient demographics and characteristics at baseline (all enrolled population)

	**All patients (N = 42)**
**Demographics:**	
Age, years, mean ± SD	45.1 ± 12.7
Gender, male: female, n	22: 20
Body weight, kg, mean ± SD	76.7 ± 16.1
Height in cm, mean ± SD	169.9 ± 9.0
Race, n (%):	
Caucasian	38 (90.5)
Asian	1 (2.4)
Hispanic	2 (4.8)
Other	1 (2.4)
**Treatment history, mean ± SD:**	
Age at diagnosis, years	24.8 ± 15.0
Age at enzyme therapy initiation, years	35.6 ± 12.5
Duration of enzyme therapy, years	9.5 ± 4.0
Duration of current dose of enzyme therapy, years*	4.9 ± 2.8
Enzyme therapy dose at switch, IU/kg/month	61.3 ± 35.7
**Disease parameters, mean ± SD:**	
Liver volume in mL (n = 40)	1726 ± 459.6
Liver volume in MN (n = 40)	0.91 ± 0.22
Spleen volume^†^ in mL (n = 30)	484.7 ± 333.2
Spleen volume^†^ in MN (n = 30)	3.13 ± 1.76
Hemoglobin, g/dL (n = 41)	14.89 ± 1.49
Platelet count, × 10^9^/L (n = 41)	193.8 ± 92.06

*Median (range) dose of enzyme therapy 54.7 (5.2–130.7) IU/kg/month; ^†^excludes splenectomized patients; MN, multiples of normal.

Patients in the ‘all enrolled’ patient population displayed relatively mild disease at baseline, with normal mean (SD) liver volume (0.91 [0.22] multiples of normal [15]), only mild splenomegaly among non-splenectomized patients (3.13 [1.76] multiples of normal [15]), and normal hemoglobin concentrations and platelet counts (Table 1). Overall, 80% of patients had attained the therapeutic goals at baseline [28]. Most patients (33/42; 78.6%) had intact spleens.

**Figure 1:**
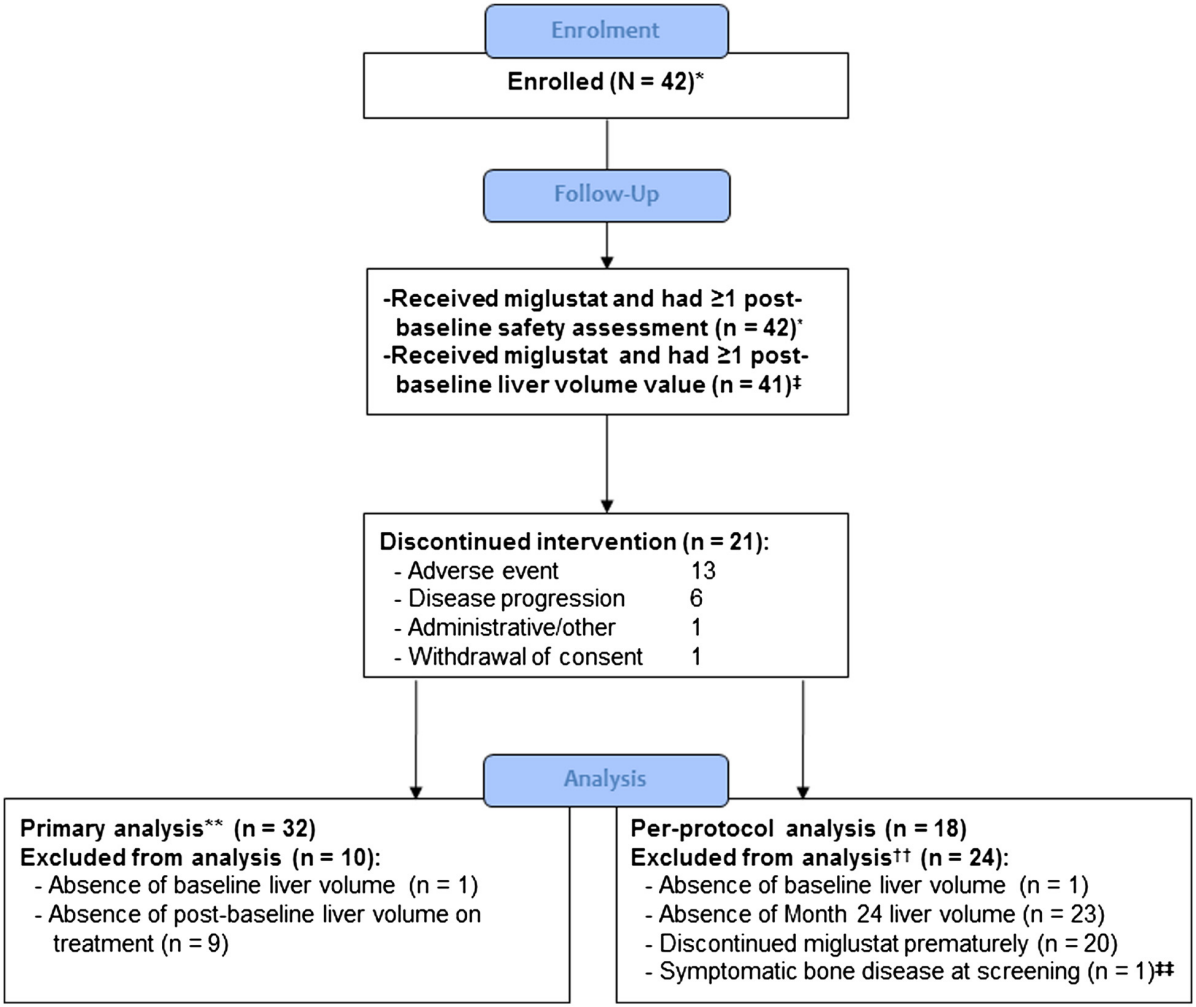
Patient disposition.^*^All enrolled population; ^‡^full analysis population; ^**^all patients with at least one post-baseline liver assessment (≥6 months) were included in primary endpoint analysis, including 11 patients who discontinued miglustat treatment prematurely; ^††^some patients had more than one of the listed reasons for non-inclusion in the per-protocol analysis; ^‡‡^Patient had documented symptomatic bone disease at screening and represented a protocol violation. Note: secondary efficacy evaluations based on full analysis population included different numbers of patients subject to data availability.

All 42 enrolled patients received ≥1 dose of the study drug (‘all-enrolled population’) and 41 patients had ≥1 post-baseline assessment for the primary endpoint (full analysis population) (Figure 1). There were a total of 21 premature discontinuations from therapy during the study, most of which were due to adverse events (13 patients); one patient withdrew for administrative reasons and one patient withdrew consent. Six patients discontinued miglustat prematurely because of disease worsening as assessed on the basis of local laboratory and data.

The median (range) exposure to miglustat treatment in the all-enrolled patient population was 658 (3–765) days. Twenty-two of the 42 enrolled patients (52%) were treated for ≥18 months. Overall, 21 patients completed 24 months of treatment, among whom 18 met criteria for inclusion in the per protocol analysis. Patient characteristics in the full analysis (n = 41) and per protocol (n = 18) patient populations were comparable with those in the entire study population.

### Primary efficacy

**Figure 2:**
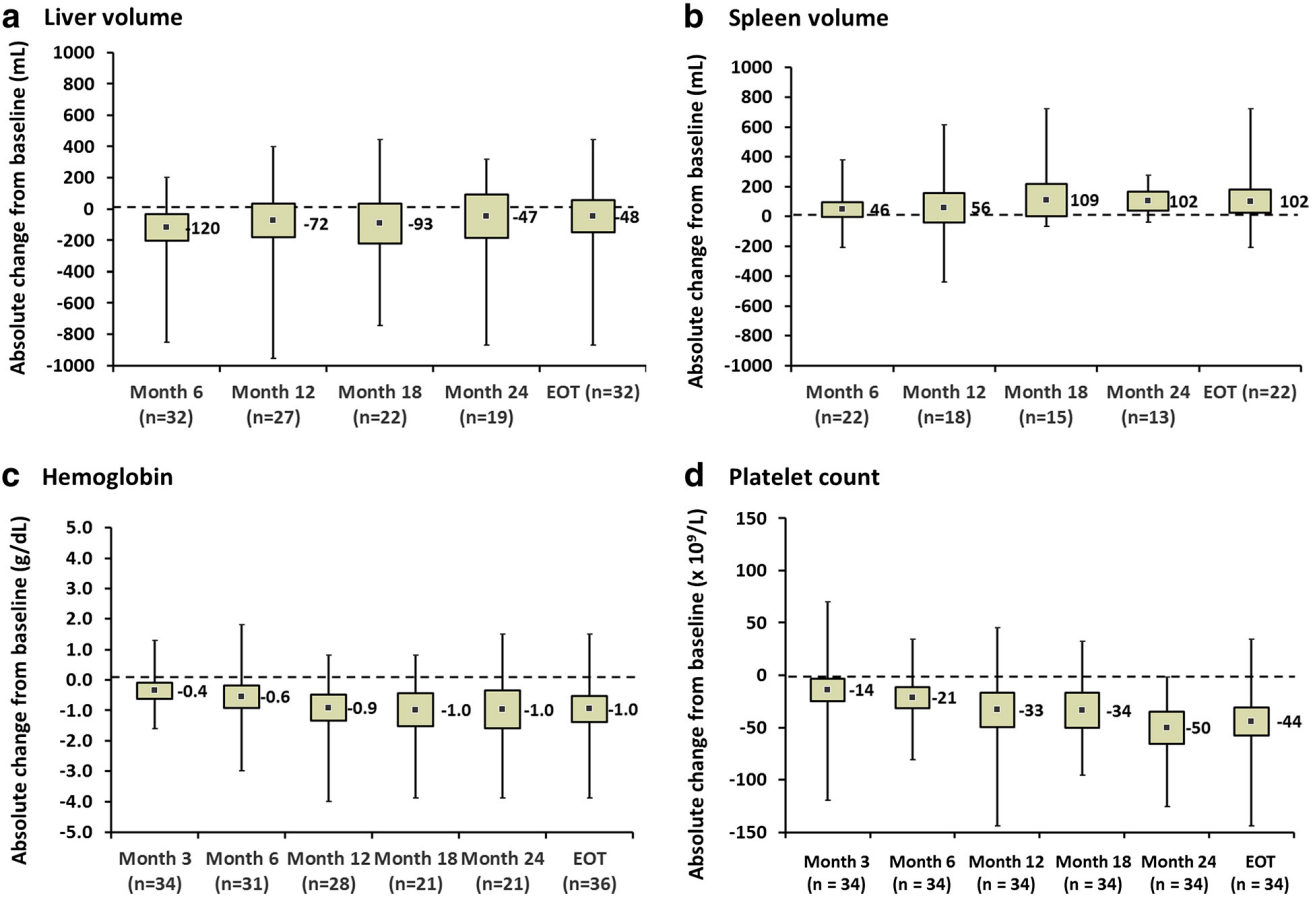
**Absolute changes in primary and secondary efficacy parameters during 24 months of miglustat therapy (full analysis population).** Changes from baseline in **a**) liver volume, **b**) spleen volume, **c**) hemoglobin concentration and **d**) platelet count. Data are mean absolute change from baseline (square points), with 95% confidence intervals (grey boxes) and minimum–maximum value ranges (solid vertical lines).

Based on patients with available data in the full analysis population (n = 32), the mean (95% CI) percent change in centrally read liver volume from baseline to end of treatment was −1.1% (−6.0% to 3.9%) (Table 2). As the null hypothesis was rejected, the primary efficacy endpoint of the study was met – the upper limit of the two-sided 95% CI was below the inferiority limit of 10%. Sensitivity analysis showed consistent findings between the full analysis population and per-protocol population for liver volume (mean [95% CI] percent change +0.1% [−7.4%, 7.5%]; n = 18). Mean absolute changes in liver volume from baseline were negligible during the course of miglustat therapy (Figure 2A).

**Table 2:** Baseline and change from baseline in liver volume during up to 24 months of miglustat therapy (primary analysis)

	**Non-splenectomized**	**Splenectomized**	**All**
	**(N = 23)**	**(N = 9)**	**(N = 32)**
Baseline absolute value (mL)			
Mean ± SD	1742 ± 440	1857 ± 604	1775 ± 484
End of treatment absolute value (mL)			
Mean ± SD	1736 ± 353	1704 ± 470	1727 ± 382
Absolute change from baseline (mL)			
Mean	−6.4	−152.6	−47.5
95% CI	(−124, 111)	(−395, 90)	(−151, 56)
Percent change from baseline			
Mean	1.3	−7.2	−1.1
95% CI	(−4.6%, 7.3%)	(−16.6%, 2.2%)	(−6.0%, 3.9%)

Data analysis based on full analysis population.

The primary efficacy endpoint was achieved in both the splenectomized and non-splenectomized patient subgroups (Table 2). Mean absolute liver volumes in these subgroups remained below baseline throughout treatment.

### Secondary efficacy

Analyses in this section are based on available data for the full analysis population. Mean absolute changes from baseline in spleen volume, hemoglobin concentration and platelet count up to Month 24 of miglustat therapy are summarized in Figures 2B–D. Individual patient efficacy findings are listed against gender and body weight in Table 3.

**Table 3:** Individual patient data and primary and secondary efficacy parameter findings

**Pt. #**	**Gender**	**Genotype**	**Body wt. (kg)**		**Tx duration (days)**	**Liver (MN)**		**Liver volume (mL)**		**Spleen (MN)**		**Spleen volume (mL)**		**Hb (g/dL)**		**Platelets (x 10**^**9**^**/l)**		Chito nmol/(mL*hr)	
			**BL**	**EOT**		**BL**	**EOT**	**BL**	**EOT**	**BL**	**EOT**	**BL**	**EOT**	**BL**	**EOT**	**BL**	**EOT**	**BL**	**EOT**
1	M	N370S/unknown	102	–	71	0.55	–	1420	–	3.40	–	696	–	15.1	–	127	–	159	–
2^†,Ŧ^	M	N370S/IVS 2 + 1	79.4	79.4	148	–	0.92	–	1833	–	5.52	–	876	15.4	15.6	93	95	781	1396
3^†^	F	R496H/G202R	59.8	57.4	734	1.05	0.94	1564	1345	SPX	–	SPX	–	13.8	13.4	613	521	2915	1881
4^†^	F	N370S/L444P	126	86.8	716	0.64	0.87	1999	1890	SPX	–	SPX	–	12.9	14.4	437	311	–	151
5^†,Ŧ^	F	N370S/unknown	69.7	62.8	408	0.85	0.85	1474	1333	2.66	3.54	371	445	14.0	12.1	233	148	974	3456
6	F	N370S/L444P	86.3	–	75	0.73	–	1582	–	2.94	–	507	–	16.8	16.9	173	173	1165	–
7^†^	M	N370S/L444P	85.0	79.5	663	0.73	0.80	1545	1593	2.19	2.68	373	425	15.5	15.6	162	127	4729	1808
8^†,Ŧ^	F	N370S/EXG DEL G	48.0	46.0	245	1.49	1.54	1785	1774	SPX	–	SPX	–	12.9	12.3	161	195	2887	5196
9^†^	M	N370S/L444P	91.0	85.0	732	0.98	0.89	2224	1891	9.90	10.4	1801	1761	14.3	14.0	100	98	1619	3139
10	M	N370S/N370S	71.0	–	50	–	–	–	–	–	–	–	–	15.3	–	139	–	–	–
11	M	N370S/N370S	70.5	69.7	729	0.98	0.92	1729	1600	–	3.12	–	435	14.0	12.7	167	100	–	2156
12^†,Ŧ^	F	N370S/T134P	57.0	46.7	373	0.77	0.80	1100	935	SPX	–	SPX	–	13.9	11.6	271	127	–	862
13	M	N370S/N370S	76.3	–	3	0.94	–	1798	–	2.21	–	338	–	16.1	–	187	–	53	–
14^†,Ŧ^	M	N370S/K198E	70.1	70.1	190	1.04	1.11	1827	1938	2.72	3.51	382	492	15.2	12.2	160	150	–	4978
15^†,Ŧ^	M	N370S/84GG	83.6	75.3	515	1.49	1.20	3112	2258	4.66	3.77	779	568	13.3	12.4	199	149	–	20
16*^,†,Ŧ^	M	N370S/S107L	78.5	72.3	405	0.83	0.91	1627	1637	SPX	–	SPX	–	13.4	11.6	283	188	420	3645
17	F	N370S/D409H	81.2	–	109	1.02	–	2062	–	4.30	–	699	–	13.4	–	106	–	839	–
18*^,†^	F	N370S/L444P	59.2	57.4	741	1.33	1.19	1964	1715	2.91	3.11	344	357	13.5	12.1	177	99	1232	4113
19^†^	M	N370S/84GG	74.7	69.6	743	0.78	0.72	1463	1254	SPX	–	SPX	–	17.0	13.1	225	155	1336	4200
20^Ŧ^	F	N37O/NK	51.5	–	112	1.04	–	1341	–	3.11	–	320	–	16.8	16.8	183	152	591	695
21^†^	F	N370S/N370S	73.5	69.0	729	0.66	0.67	1220	1148	1.44	1.29	211	179	13.8	13.3	160	153	80	41
22*^,†^	F	N370S/crossover	62.5	58.2	755	1.00	1.16	1562	1687	3.57	5.11	447	595	16.4	15.4	167	111	2326	2969
23^†^	F	N370S/unknown	82.5	68.5	718	0.93	1.00	1925	1706	5.03	6.40	830	877	16.6	16.1	104	91	389	4058
24^†^	F	N370S/N370S	67.7	65.9	740	0.82	0.86	1391	1416	2.00	1.80	270	238	16.4	14.6	226	191	27	17
25*^,†^	M	N370S/1327TT	72.0	72.0	754	1.22	1.22	2191	2204	4.51	6.23	650	897	15.0	14.8	152	79	2658	8348
26*^,†^	F	N370S/REC NEIL	61.0	51.0	732	0.95	1.06	1444	1349	1.61	2.84	197	289	14.8	13.4	186	113	666	3500
27*^,†,Ŧ^	F	N370S/L444P	55.9	56.8	478	1.12	1.25	1571	1770	4.01	5.08	449	577	13.6	10.8	148	99	1872	2897
28^†,Ŧ^	M	N370S/1035INSG	93.7	87.7	545	1.02	1.15	2391	2530	1.43	1.94	268	340	11.3	11.1	151	155	5863	6772
29*^,†^	M	ND	91.2	87.3	653	0.81	1.05	1847	2291	6.67	11.1	1217	1939	15.0	15.1	98	54	241	1703
30*^,†^	M	ND	86.1	84.8	765	0.81	0.97	1745	2063	2.40	4.07	413	690	16.8	14.7	158	116	3398	7138
31^†^	M	N370S/N370S	95.9	82.3	734	0.82	0.90	1964	1854	SPX	–	SPX	–	14.8	14.4	264	251	–	110
32^†,Ŧ^	M	N370S/8499	71.2	79.8	239	0.92	0.75	1638	1496	2.18	2.18	311	348	14.9	14.1	186	128	391	590
33^†^	F	N370S/R257X	124	109	738	1.05	0.88	3274	2400	SPX	–	SPX	–	17.8	13.9	229	219	44	1071
34*^,†^	F	N370S/unidentified	113	109	725	0.63	0.64	1788	1733	1.74	2.96	394	642	13.6	14.6	223	135	1587	4118
35*^,†^	F	N370S/unidentified	89.2	68.8	725	0.52	0.85	1152	1468	2.23	3.24	398	446	15.3	14.8	149	102	132	1002
36*^,†^	M	N370S/unidentified	70.3	68.9	725	0.97	1.09	1704	1880	2.94	4.34	413	598	14.2	14.6	177	158		13834
37^Ŧ^	M	N370S/unknown	76.0	–	140	0.75	–	1434	–	2.11	–	320	–	14.7	14.4	141	141	438	510
38*^,†^	F	84GG/N370S	65.0	62.5	732	0.72	0.92	1162	1439	3.24	3.72	422	465	16.8	15.1	136	106	98	1271
39*^,†^	M	N370S/84GG	88.0	81.0	730	0.88	1.11	1932	2248	SPX	–	SPX	–	17.1	14.8	254	174	1940	10636
40	M	L444P/NA	54.4	–	22	0.81	–	1095	–	2.18	–	238	–	16.2	–	134	–	–	–
41	M	N370S/N370S	76.7	–	15	0.80	–	1538	–	1.37	–	210	–	15.9	–	232	–	–	38
42^†,Ŧ^	F	N370S/N370S	58.5	58.0	231	1.01	0.98	1471	1424	2.35	2.54	275	294	12.6	12.9	214	207	17	21

BL, baseline; Chito, chitotriosidase; EOT, end of treatment; Hb, hemoglobin; MN, multiples of normal; ND, not determined; SPX, splenectomy; Tx, treatment; WV, worst value. *Patients adjudicated as showing disease worsening; ^†^patients within therapeutic goals at baseline who were assessed for maintenance of therapeutic goals; ^Ŧ^patients in whom therapeutic goals were adjudicated based on worst value (end-of-treatment) analysis. Dashes represent patients in whom useable data values were not available or analyses (i.e. therapeutic goals) were not possible.

Overall, spleen volume among the 22 evaluable, non-splenectomized patients showed a mean (95% CI) increase of 102 (24, 180) mL by Month 24 from a mean (SD) baseline value of 510 (372) mL; the mean percent change by Month 24 was 23% (95% CI: 7, 39). Changes from baseline appeared maximal by Month 18.

There was a small mean (95% CI) decrease in hemoglobin concentration of 0.95 (−1.38, −0.53) g/dL at Month 24, from a mean (SD) baseline value of 14.8 (1.53) g/dL. The mean change from baseline was maximal by Month 18, and the mean (SD) hemoglobin concentration was 13.9 (1.55) g/dL at end of treatment.

Platelet count showed a mean (95% CI) decrease of 44 (−58, −31) × 10^9^/L from a mean (SD) baseline value of 199 (96) × 10^9^/L. Again, maximal changes from baseline were observed at Month 18. Baseline platelet counts were higher in splenectomized patients (mean [SD] 304 [137] × 10^9^/L) than in non-splenectomized patients (mean [SD] 164 [38] × 10^9^/L). Mean (95% CI) decreases in platelet counts from baseline to Month 24 were also greater in splenectomized patients (−66 [−111, −21] × 10^9^/L) compared with the non-splenectomized subgroup (−37 [−48, −25] × 10^9^/L). There were no reported episodes (adverse events) of bleeding or bruising in either subgroup during miglustat therapy.

Sensitivity analysis demonstrated consistent findings between the per-protocol population and the full analysis population for each key secondary endpoint.

Total BMB score evaluations from baseline to end of treatment were available from 21 patients in the full analysis population (15 non-splenectomized and 6 splenectomized). Overall, BMB scores showed a slight increase (worsening) during the study; the median (95% CI) absolute total BMB score increased from 5 (3, 7) at baseline to 7 (4, 11) at the end of treatment – a median (95% CI) increase of 3 (0, 4). Eleven patients showed an increase of ≥3 points. There was no change in median (95% CI) BMB score in splenectomized patients (0 [−3, 5]; n = 6) at the end of treatment compared with a median (95% CI) increase of 3 (0, 7) among non-splenectomized patients (n = 15). These results are consistent with an increase in Gaucher cell infiltration in the bone marrow in some of the splenectomized patients.

Plasma chitotriosidase activity determinations at baseline and end of treatment were available for 28 patients. Chitotriosidase activity showed marked inter-individual variability at baseline, despite all patients having mild, stable disease (range 17–5,863 nmoles/[mL*hr]). From a baseline median (95% CI) value of 963 (420, 1,872) nmoles/(mL*hr), there was a median (95% CI) increase of 1,173 (643, 2,834) nmoles/(mL*hr) at end of treatment. Analysis of changes in chitotriosidase activities between baseline and end of treatment for each patient revealed a median (range) within-patient percent change of +102% (−62% to 2334%).

A total of 20 cases were referred to the Steering Committee for adjudication of potential disease worsening, including 11 patients referred during the study and 9 cases referred at the final end-of-study adjudication. Thirteen patients were confirmed as having disease worsening. For the remaining seven patients who were referred with one or more signals of possible worsening, when considered alongside the overall clinical picture of the patient, together with the clinical relevance of the findings, the Steering Committee did not consider these particular signals sufficient to constitute disease worsening. On the basis of local assessment of organ volume, two of these seven patients had been adjudicated as worsened during the study; however, on the basis of standardized central readings, they were not confirmed as worsened at the end-of-study adjudication (patients 5 and 8 in Table 3).

### Maintenance of therapeutic goals

Most patients in the full analysis population who were within therapeutic goals for key type 1 Gaucher disease parameters at baseline were maintained within goal up to Month 24. Thirty-two patients had a baseline and a post-baseline assessment for liver volume while on miglustat therapy, and 29/32 (91%) who were within goal at baseline remained within goal at Month 24. Of the three patients who were outside the goal at baseline, two were within the goal and one remained outside goal by the end of treatment. For other parameters, the numbers and proportions of patients with available data who were within goal at baseline and remained within goal at the end of treatment were: 20/21 (95%) for spleen volume; 33/35 (94%) for hemoglobin concentration; 29/34 (85%) for platelet count. Overall, 21/25 (84%) evaluable patients showed maintenance of disease stability across all disease parameters between baseline and the end of treatment.

### Safety and tolerability

Safety and tolerability assessments did not reveal any new or unexpected safety findings, and were consistent with the known safety profile for miglustat [11]. Gastrointestinal disorders were the most frequently reported adverse events among the all-enrolled population, with diarrhea, flatulence and abdominal discomfort reported most frequently (Table 4). Eleven out of 41 patients (27%) in the full analysis population commenced as-needed treatment with loperamide for gastrointestinal disturbances during miglustat therapy.

**Table 4:** Treatment-emergent adverse events* during 24 months of miglustat therapy (all-enrolled population)

**Adverse event**	**Incidence (N = 42)**	
	**n**	**%**
Diarrhea	31	31 (74)
Flatulence	21	21 (50)
Tremor	15	15 (36)
Headache	9	9 (21)
Paresthesia	9	9 (21)
Fatigue	8	8 (19)
Dizziness	7	7 (17)
Arthralgia	6	6 (14)
Weight decreased	6	6 (14)
Abdominal discomfort	5	5 (12)
Hypoesthesia	5	5 (12)

*Adverse events with ≥10% incidence.

Body weight decrease was reported as an adverse event in 6/42 (14.2%) patients, among whom 5 cases were considered related to miglustat therapy. The mean (SD) decrease in body weight among all patients between baseline and Month 24 was –7.7 (9.1) kg; median (range) decrease –4.6 (−39.0–0) kg.

Doses of miglustat were reduced from 100 mg t.i.d. to 100 mg b.i.d. in a total of three patients due to adverse events: two were due to gastrointestinal disturbances and one was due to cerebellar syndrome and hyper-reflexia. Two of these patients later discontinued miglustat treatment prematurely, and their adverse events resolved with no clinical sequelae. One patient (with dose reduction due to diarrhea) went on to complete the study.

Thirteen out of 42 (31%) patients in the all-enrolled population discontinued miglustat therapy on account of adverse events that did not indicate worsening of their disease. Diarrhea and tremor were associated with discontinuations in 7/42 (17%) and 3/42 (7%) patients, respectively. Abdominal discomfort and abdominal distension were associated with discontinuations in 1 patient each. Most discontinuations related to adverse events occurred early during miglustat therapy: eight patients discontinued miglustat before Month 6, and two others between Months 6 and 12.

A further 5/42 (11.9%) patients discontinued miglustat therapy prematurely due to adverse events that may have been indicative of disease worsening according to protocol-defined criteria. These included increased chitotriosidase activity, decreased platelet count, decreased hemoglobin and splenomegaly. The reason for early discontinuation of miglustat therapy in these patients was recorded as ‘disease worsening’ in accordance with the study protocol in order to differentiate discontinuations due to tolerability issues from those required by the disease worsening criteria (see *Secondary efficacy* section); one patient was discontinued due to ‘disease worsening’ criteria without any corresponding adverse event being recorded.

All 42 patients had a baseline electrodiagnostic assessment, among whom three (7.1%) were diagnosed with mononeuropathy. Seven (16.7%) had minor electrodiagnostic abnormalities without clinical evidence of peripheral neuropathy, and five (12%) had electrodiagnostic abnormalities compatible with peripheral neuropathy. During the study, one of the seven patients with minor electrodiagnostic abnormalities but no clinical signs or symptoms of peripheral neuropathy at baseline developed a mild sensorimotor neuropathy that was not attributed to the study medication. This patient was found to have vitamin B_12_ deficiency, which was treated, and miglustat was continued without ill effect.

Neuropsychological investigations indicated no signs of cognitive deterioration associated with miglustat treatment. There was no consistent or progressive decline among the various neurocognitive tests.

## Discussion

Oral treatment with miglustat for 24 months prevented pathological storage in liver as evidenced by the maintenance of liver size. The mean percent change in liver volume from baseline during 24 months of miglustat therapy (−1.1) was well within the pre-defined non-inferiority limit of 10%, and thus the primary efficacy endpoint of this 24-month, open-label non-inferiority study was met. In addition, all patients who were within the proposed therapeutic goal for liver volume at baseline were maintained within this goal throughout the study [21]. Liver volume was therefore maintained in patients with stable type 1 Gaucher disease who changed to miglustat after previous long-term enzyme therapy.

Change in liver volume has been recommended as a consistent clinical indicator of disease course and treatment effects in type 1 Gaucher disease as it does not tend to show the degree of inter-patient variability seen with other key disease parameters [28]. Liver volume or ratio has been assessed as the primary efficacy endpoint in a number of previous clinical trials alone or in combination with spleen volume [15,19,29], and was chosen following detailed discussions with the EMA at the time of trial design. Moreover, the analytical approach adopted in this study allowed an objective, quantitative assessment of this endpoint.

Inclusion of a placebo arm was considered unethical and impracticable in this treatment and patient setting, which is unfortunate as the absence of a suitable control group prevents a robust evaluation of the effects of miglustat in patients who have already had the benefit of enzyme therapy. Previous data indicate that a 2-year treatment interruption would carry a risk of recurrent clinical disease in some patients, accompanied by severe fatigue and reappearance of bone pain and other skeletal manifestations of untreated Gaucher disease [10,30-33].

Exploratory analysis of the key secondary efficacy endpoints (spleen volume, hemoglobin concentration and platelet count) were included in the trial design after the primary endpoint was agreed with the EMA, based on their scientific relevance. Overall, these secondary endpoints indicated stable disease between baseline and the end of miglustat treatment. High proportions of patients who were within therapeutic goals for each parameter at baseline were maintained within goal up to Month 24.

The clinical relevance of the absolute mean changes in secondary efficacy parameters, most of them in the direction of disease progression, is difficult to interpret. The mild baseline disease burden of the study population should be taken into account. For instance, the 102 mL increase in spleen volume in non-splenectomized patients at end of treatment should be considered in the context of the low mean absolute baseline value of 510 mL, compared with values normally seen in long-term enzyme therapy patients. In all, 8/30 (27%) patients showed clinically relevant increases in spleen volume.

Small mean absolute reductions in hemoglobin were observed during miglustat therapy(−0.95 g/dL from a baseline of 14.8 g/dL). Four out of 41 (10%) patients had clinically relevant decreases in hemoglobin indicative of disease progression but, as with the organ volume findings, the great majority (94%) of patients was maintained within the normal range. The proportion of patients remaining stable for platelet count (85%) from baseline to Month 24 was slightly lower than for other key parameters, with an absolute mean change of −44.1 × 10^9^/L. Nine of 41 (22%) patients had decreased platelet counts that were considered potentially relevant.

The relevance of plasma chitotriosidase for monitoring disease status in patients with stable, mild type 1 Gaucher disease after switching to miglustat in relation to prognosis is currently unclear. Consistent with other systemic disease parameters, the median absolute chitotriosidase activity was low at baseline (963 nmoles/[mL*hr]) but was variable between patients despite long-term enzyme therapy. Changes in chitotriosidase activity in response to miglustat treatment were also highly variable, with some patients showing marked increases; this suggests that these patients had ‘subclinical’ disease progression, which was unassociated with clinical events. Miglustat has been shown to significantly reduce plasma chitotriosidase activity in treatment-naïve patients over 3 years of therapy [13,14]. In the previous open-label randomized study reported by Elstein et al. [19], mean chitotriosidase activity remained stable during miglustat therapy in 27/28 (96%) patients with type 1 Gaucher disease after switching from long-term enzyme therapy. Similar findings were reported in the Spanish ZAGAL cohort study [16].

Bone pain (rated on a visual analogue scale ranging from 0 to 100) was unchanged during 24 months of miglustat treatment, and none of the 17 evaluable patients who were free of bone pain at baseline experienced pain episodes throughout follow up. MRI-based BMB scores indicated a slight increase in bone marrow infiltration over 24 months. Again, this may indicate subclinical disease progression in some patients. Of note, there was no median change in BMB score among the six splenectomized patients, compared with the small median change (+3) among the 15 patients with intact spleens. Previously, stable BMB scores have been observed for up to 18 months in patients changed from enzyme therapy to miglustat [34]. Long-term follow up in a Spanish cohort of switch patients showed an improvement in lumbar spine bone marrow infiltration during 48 months of therapy [16].

It is not possible to discern whether patient genotype had an influence on the changes in these disease parameters, due to the small sample size. From the genotype data presented in Table 3 there does not appear to be any clear association of a particular genotype with disease worsening or improvement during miglustat therapy.

To ensure the safety of patients who might show deterioration, we used conservative exploratory criteria to identify patients with ‘suspected disease worsening’ after switching from a stable dose of enzyme therapy to miglustat. These criteria do not reflect those generally used in clinical practice [35]. Based on the criteria defined in the trial protocol, miglustat treatment was discontinued in a total of six patients, as adjudicated by the Steering Committee. The number of patients finally adjudicated as exhibiting disease worsening (n = 13) was lower than the total number of cases with suspected disease worsening referred by investigators during or at the end of the study (n = 20). This may be related to the fact that decision making regarding disease worsening during the study was based on local readings, while the Steering Committee utilized both local readings and standardized central readings at the end of the study.

No new or unexpected safety findings came to light during this 24-month study and, overall, the safety and tolerability of miglustat was found to be consistent with its known safety profile [11,13-15,19,36]. Gastrointestinal effects such as diarrhea and flatulence were the most frequently reported adverse events, and were the most frequent reason for premature discontinuation of therapy. In this respect, it is regrettable that firm guidance for avoiding or ameliorating gastrointestinal disturbances, such as dietary modification in the early phase of treatment, was not specified in the study protocol. Such interventions during the initial weeks of miglustat therapy can substantially improve tolerability [37,38]. Notably, patients attending the one center where individual dietary guidance *was* provided reported the least severe gastrointestinal side effects.

No new cases of peripheral neuropathy with clinical symptoms attributable to miglustat occurred in the present study. The observed 11.9% prevalence of neuropathy at baseline is in agreement with the prevalence of peripheral neuropathy (11%) reported in a previous study of 103 type 1 Gaucher disease patients not receiving this agent [4]. Findings from the neuropsychological assessments conducted in this study were in line with data from the miglustat post-marketing surveillance program [36].

To an extent, the current study allows a limited interpretation of the therapeutic role of miglustat in Gaucher disease. The wide variability in response between patients included in this study, alongside the appreciable rate of premature discontinuations from therapy, suggest that the use of miglustat as a maintenance therapy in GD1 might be suitable for a distinct group of patients. However, additional data would be required to fully define such a group.

With regard to the higher than expected patient dropout from this study, it seems likely that the stringent criteria we adopted to define ‘disease worsening’ may have contributed to their withdrawal. However, we defend the rigorous adjudication process because of the need to ensure that no patient experienced unnecessary or untoward deterioration during the protracted (2-year) course of the trial. The criteria that were adopted for withdrawing patients from this study do not reflect those that are used in therapeutic decision-making regarding treatment failure in clinical practice. Finally, while an examination of the achievement of therapeutic goals was included in the statistical analysis plan, it was not defined *a priori*, and thus should be interpreted with caution. In addition, such an analysis may lack sensitivity, as it is possible that patients experiencing deterioration in a particular disease parameter could still remain within the range for the goal.

## Conclusions

The primary efficacy endpoint was met; overall there was no change in liver volume during 24 months of miglustat therapy. However, a notable proportion of patients showed a gradual deterioration in some disease manifestations, suggesting that miglustat could maintain clinical stability, but not in all patients. Miglustat demonstrated a predictable profile of safety and tolerability that was consistent with that reported in previous clinical trials and experience in clinical practice. Gastrointestinal tolerability was identified as an important clinical management issue in this study and should be actively addressed, particularly during the early weeks of miglustat therapy.

## Appendix

*Miglustat Maintenance Study Group investigators and patient numbers per center:* Amato D, Toronto, Canada (n = 5); Belmatoug N, Paris, France (n = 1); Cox TM, Cambridge, UK (n = 6); Fernhoff P, Decatur, USA (n = 3); Giraldo P, Zaragoza, Spain (n = 2); Giugliani R, Porto Alegre, Brazil (n = 1); Hollak CEM, Amsterdam, the Netherlands (n = 3); Hwu W-L, Taipei, Taiwan (n = 1); Malinová V, Prague, Czech Republic (n = 2); Packman S, San Francisco, USA (n = 1); Pastores G, New York, USA (n = 3); Rhead WJ, Wauwatosa, USA (n = 3); Rowell J, Herston, Australia (n = 1); Steiner RD, Oregon, USA (n = 6); Szer J, Carlton, Australia (n = 2); Tifft-Rosenbaum K, Washington DC, USA (n = 2).

## Competing interests

TMC has received unrestricted research funding from Cambridge in America, Genzyme Corporation and Shire Human Genetic Therapies, and has received lecturing and conference fees (not retained) from Genzyme Corporation, Shire Human Genetic Therapies and Actelion Pharmaceuticals. He has also received honoraria for investor consultancy from Genzyme Corporation, and has provided expert advice on legal proceedings related to neglected treatment of acute porphyria. DA has received research and operating grants, honoraria for speaking, and travel funds from Actelion Pharmaceuticals, Genzyme Corporation, Protalix Biopharmaceuticals, and Shire Human Genetic Therapies. CEMH has received consultancy fees from Actelion for participation in clinical trial programs and other projects, and speaker fees for participation in scientific congresses and sponsored events. CEMH donates all fees to the Gaucher Stichting, a national foundation that supports research in the field of lysosomal storage disorders. RDS has received research grants from TKT/Shire, honoraria for speaking and consulting, travel funds from Actelion Pharmaceuticals, and honoraria from Genzyme, Biomarin, Amicus, and Shire Human Genetic Therapies. CL is an employee and stockholder at Actelion. MS and RG were employees at Actelion at the time this research was performed.

## Authors’ contributions

TMC was the principal clinical investigator. DA, CEMH and RDS were participating clinical investigators. All clinical investigators were involved in the recruitment and treatment of patients and the provision of raw clinical data. RG was the International Clinical Leader of the trial at Actelion, providing oversight of all aspects of trial conduct and data analyses. CL was the Scientific trial leader at Actelion and provided overall trial management. MS at Actelion planned and conducted the statistical analysis of the trial data. All authors have reviewed and interpreted the data, reviewed each draft of the manuscript and approved the final version for submission.
